# Current progress and role of lncRNAs in HBV infection and progression of hepatocellular carcinoma

**DOI:** 10.1016/j.gendis.2025.101878

**Published:** 2025-10-13

**Authors:** Faisal Mahmood, Maher Un Nisa Awan, Meng-Ting Luo, Xiao Li, Xiao-bin Peng, Jun Xu, Jia Wei, Tai-Cheng Zhou

**Affiliations:** a Central Laboratory, The Affiliated Hospital of Yunnan University, Kunming, Yunnan 650021, China; b Department of Neurology, The Affiliated Hospital of Yunnan University, Kunming, Yunnan 650021, China; c School of Medicine, Yunnan University, Kunming, Yunnan 650091, China; d Department of Neurology, Beijing Tiantan Hospital, Capital Medical University, China National Clinical Research Center for Neurological Diseases, Beijing 100070, China; e Department of Neurology, The Second People's Hospital of Guiyang (Jinyang Hospital), The Affiliated Jinyang Hospital of Guizhou Medical University, Guiyang, Guizhou 550081, China

**Keywords:** Hepatitis B virus, Hepatocellular carcinoma, Immune dysfunction, Long noncoding RNAs, Virus

## Abstract

Infection with hepatitis B virus (HBV) remains a severe concern to public health, with roughly 292 million people worldwide suffering from the chronic form of the disease, for which there is no cure. Chronic HBV infections frequently lead to hepatocellular carcinoma (HCC), one of the world's leading causes of cancer-related deaths. Although the process of hepatocarcinogenesis is complex and not fully understood, various studies have identified numerous long non-coding RNAs (lncRNAs) as contributing to the formation of HCC. These host-derived lncRNAs are frequently dysregulated as a result of viral infection. Numerous lncRNAs have been linked to HBV carcinogenesis and replication, particularly those that are dysregulated in HBV-associated HCC. HBV X protein regulates the majority of these dysregulated lncRNAs. Certain lncRNAs have been found to exert regulatory functions in HBV replication and carcinogenesis. The prognosis for HCC remains poor, and early detection of novel tumor markers is critical for effective HCC therapy. Understanding the biological activities and regulatory mechanisms of HCC-associated lncRNAs will aid in disease diagnosis and therapy and help elucidate the disease etiology. In HBV-related HCC, certain dysregulated lncRNAs may develop into biomarkers for early detection or potential targets for HCC treatment. This review provides a brief overview of the recent findings on lncRNAs in HBV with a focus on current developments. We also investigated the possible relevance of dysregulated lncRNAs in HCC as biomarkers for diagnosis and treatment and assessed their carcinogenic and tumor-suppressive effects.

## Introduction

**Figure 1:**
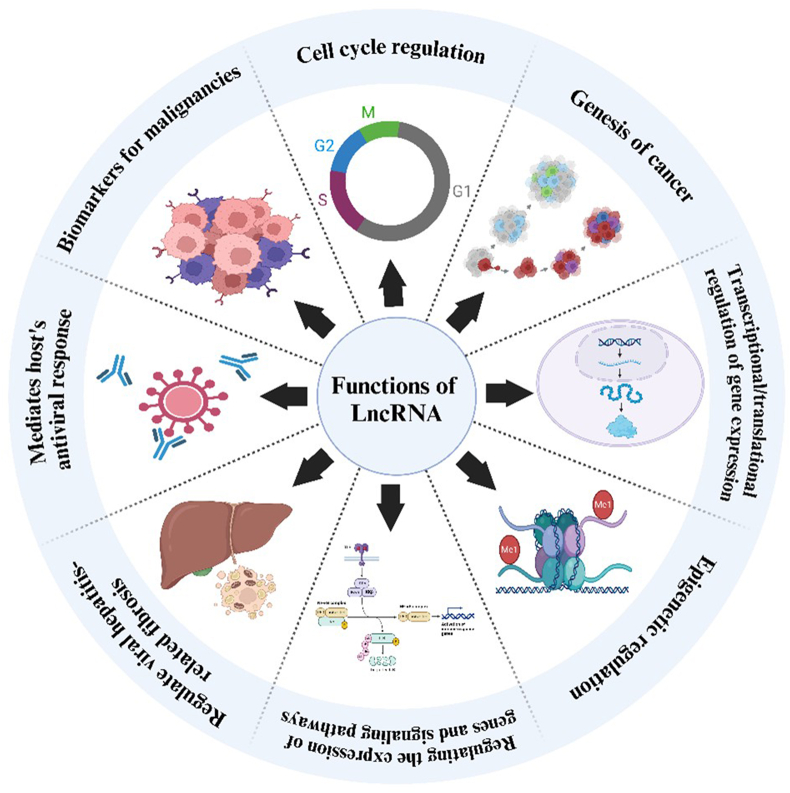
Involvement of lncRNAs in different biological functions.

Long non-coding RNAs (lncRNAs) are non-coding RNAs that are longer than 200 nucleotides. They regulate a wide range of physiological and pathological processes.^1^^,^^2^ To distinguish between long and short noncoding RNAs, RNA purification processes were first used to determine a cut-off length of 200 nucleotides. However, the difference in this length is insufficient to adequately explain the functional features of noncoding RNAs.^3^^,^^4^ The majority of the known lncRNAs are transcriptionally controlled by RNA polymerase II. These transcripts are found in the cytoplasm or nucleus and may be polyadenylated.^5^ Although lncRNAs are thought to be more abundant in the genome than protein-coding genes, there are currently no accurate and comprehensive classification and identification systems. lncRNAs are classified according to their position in relation to protein-coding genes, as intergenic, sense, and antisense (exonic, intronic, or overlapping), and bidirectional.^6^ lncRNAs are categorized as RNAs according to their functions, which include protein activity modulation, mRNA translation, gene expression, and acting as microRNA (miRNA) decoys to release target mRNAs.^7^ Numerous biological processes have been associated with lncRNAs, including cell development, differentiation, and senescence; apoptosis; cell cycle regulation; and transcriptional, post-transcriptional, and translational regulation of gene expression.^8^^,^^9^ Figure 1 illustrates the diverse functions of lncRNAs in cellular processes. Key phases of cancer development have been found to disrupt these regulatory systems and modulate lncRNA expression. Moreover, other studies have suggested the use of lncRNAs for diagnostic and therapeutic purposes, such as biomarkers for specific cancers, targets for regulating lncRNA expression, and candidates for therapeutic interventions.^10^

Although the precise role of lncRNAs in the development of cirrhosis and liver fibrosis is still unknown, emerging data suggest that numerous lncRNAs may influence these conditions through various mechanisms.^11^, ^12^, ^13^, ^14^, ^15^ The importance of lncRNAs in viral hepatitis and related liver disorders has been recently explored.^16^, ^17^, ^18^ Numerous studies have revealed lncRNA dysregulation in hepatocellular carcinoma (HCC) associated with hepatitis B virus (HBV). According to these studies, the lncRNA expression patterns of HCC and HBV-related HCC may differ. Although a number of lncRNAs have been associated with the initiation and progression of HBV-related HCC, their exact mechanisms of action are still unknown. It suggests that lncRNAs may influence the risk of HCC related to HBV in a variety of ways. The hepatitis B virus X protein (HBx) modulates many of these lncRNAs; however, more research is needed to identify the underlying regulatory mechanism.^19^ The results of many research studies exploring circulating lncRNAs in patients with HBV-related HCC have suggested that circulating lncRNAs may serve as new biomarkers for HBV-related HCC.^19^^,^^20^ Therefore, studying lncRNAs that are differentially expressed in HBV-related HCC samples may help identify new pathogenic pathways and serve as diagnostic and treatment targets.

Notably, several lncRNAs are known to serve as a bridge between chronic liver diseases and HCC. Among the lncRNAs related to both HCC and viral hepatitis-induced liver fibrosis are lncRNA ATB, HOX transcript antisense intergenic RNA (HOTAIR), lncRNA-p21, and highly up-regulated in liver cancer (HULC). These lncRNAs may be used for HCC diagnosis and prognosis, and may impede the development of HCC from chronic liver diseases.^21^ Although their role in hepatic fibrosis and cirrhosis development is still unresolved, lncRNAs influence viral hepatitis-related fibrosis and cirrhosis through a number of mechanisms.^22^ Therefore, a better understanding of the genetic changes in lncRNAs associated with liver fibrosis and cirrhosis is critical for developing new diagnostic and treatment options. Various lncRNAs have been shown to promote the genesis and progression of various liver illnesses, and therefore, studying their genetic variations may help identify potential biomarkers and therapeutic targets.

The differential expression of lncRNAs and the disruption of regulatory networks are considered crucial phases in the development of cancer. However, the role of lncRNAs in HBV infection remains unclear. HBV-infected, HBx-expressing, and HBV-related HCC cells often display dysregulated lncRNAs.^23^ lncRNAs are crucial for regulating signaling cascades and gene expression.^24^ Viral infections have been shown to influence cellular antiviral lncRNA regulation and to use lncRNAs to modulate metabolic networks to enhance virus survival. Viral protein synthesis and replication can also alter cellular lncRNA expression. lncRNAs both positively and negatively regulate the antiviral response in the innate immune system. For example, lncRNAs were shown to modulate the biological components of the innate immune response, such as natural killer cells and macrophages.^25^ Recent studies have shown that lncRNAs, including HOTAIR, maternally expressed gene 3 (MEG3), and HULC, are involved in the development of HBV-associated HCC.^26^ HBV may interfere with HOTAIR expression, which may subsequently promote tumor growth by interfering with fundamental metabolic and cell cycle regulatory processes that are necessary to maintain the high level of hepatocyte differentiation. HULC has been associated with angiogenesis and lipogenesis in hepatoma cell lines. Genes associated with proliferation have also been shown to be deregulated by siRNA knockdown of HULC. Furthermore, several studies demonstrated that inflammatory processes increased HULC and HOTAIR expression, which inhibited proliferation and promoted inflammatory responses and apoptosis.^27^ Because HOTAIR and HULC promote HBV transcription and replication, they may be considered potential new biomarkers for HBV diagnosis and treatment. Thus, elevated levels of lncRNAs in individuals on antiviral drugs may be associated with HCC.^28^

## Role of lncRNAs in viral infection and host-pathogen interactions

High-throughput transcriptome studies in humans and animals have shown that viral infection considerably affects the host cell's transcriptome, including a sizable number of lncRNAs and mRNAs.^29^^,^^30^ Based on promoter prediction and expression correlation analyses, a significant number of lncRNAs activated during viral infections were identified to target interferon (IFN) signaling or IFN-stimulated genes (ISGs). This was confirmed by experimental findings in primary human hepatocytes treated directly with IFN-α and mouse macrophages activated with recombinant IFN-β or infected with the vesicular stomatitis virus (VSV). Intriguingly, it is observed that different host lncRNAs express themselves in different ways and are induced in a matter of minutes, while others take days to express. A study on HuH7 hepatocytes showed up-regulation of almost all protein-encoding genes. The delayed timepoint of the IFN response, which occurs 72 h following high-dosage IFN-α2 treatment, was the main focus of the research.^31^ In another study, half of the identified IFN-regulated lncRNAs were up-regulated, while the other half were down-regulated.^32^ In addition to IFN-regulated lncRNAs, experimental findings from both wild-type and IFN receptor-deficient macrophages showed that viruses also stimulated certain host lncRNAs referred to as virus-hijacked lncRNAs, in which lncRNA expression did not rely on IFN signaling. These IFN-independent lncRNAs are implicated in viral invasion and are often manipulated by viruses. This includes two intergenic lncRNAs, lncRNA aconitate decarboxylase 1 (ACOD1) and virus-inducible lncRNA (VIN). Many viruses, such as VSV, Sendai virus (SeV), herpes simplex virus 1 (HSV-1), and vaccinia virus (VACV), promote the expression of lncRNA ACOD1, and this induction was shown to be partially dependent on nuclear factor kappa-light-chain-enhancer of activated B cells (NF-kB) signaling. Since interferon regulatory factor 3 (IRF3) deletion led to increased ACOD1 expression, IRF3 signaling was also inhibited, indicating that lncRNA ACOD1 is more beneficial for viruses than the host.^33^

Human lung epithelial cells have been shown to produce VIN in response to a variety of influenza A virus (IAV) strains, including H1N1, H3N2, H7N7, and VSV. However, it was shown that VIN was not produced by IFN-β therapy, RNA mimic stimulation, or influenza B viral infection, thus indicating that some viruses specifically exploited VIN over millions of years of evolution.^34^ Interestingly, it was shown that a single virus regulated a number of host RNAs in HCV infection, including CSR19, CSR21, CSR26, and CSR34, highlighting their distinct function in HCV infection.

The antiviral response and viral replication were found to be regulated by lncRNAs, which also modulate epigenetic regulation.^35^^,^^36^ Within infected cells, both viral lncRNAs and cellular lncRNAs produced by the infection are present. Viral lncRNAs have been shown to boost viral replication through autonomous replication and reduce the antiviral response through transcriptional regulation of the host and viral genomes or transcriptomes.^37^^,^^38^ Cellular lncRNAs generated during infection were found to be the primary mediators of the antiviral response. By modifying antiviral factors, such as bone marrow stromal antigen 2 IFN-stimulated positive regulator (BISPR) and nuclear enriched abundant transcript 1 (NEAT1), cellular lncRNAs have been identified to increase the antiviral response,^39^ however, lncRNAs like long non-coding RNA interleukin-7 receptor (IL7R) and heterogeneous nuclear ribonucleoprotein L-related immunoregulatory lncRNA (THRIL) inhibited the antiviral response by adversely affecting the IFN pathway.^40^^,^^41^ In addition to their function in viral infection and suppression, lncRNAs were shown to participate in various biological cellular processes, including cancer progression.^42^ Abnormally expressed lncRNAs, such as H19 and MVIH, have been found in a range of cancers, including HCC.^43^ Table 1 summarizes other lncRNAs involved in various viral infections.

**Table 1:** The regulatory roles of lncRNAs in diverse viral infections.

lncRNA	Virus	Affected signaling molecules/pathways	Mechanism of action	Reference
NEAT1	HSV-1	STAT3	The interaction between viral gene promoters and STAT3 boosted viral replication and gene expression.	44
	Human immunodeficiency virus (HIV)	Post-transcriptional activation of CD4^+^ T cells	NEAT1 negatively controlled the production of viruses in human cell lines by promoting HIV-1 transcript splicing by boosting nuclear export of INS-containing HIV-1 mRNAs. HIV-1 infection took advantage of the down-regulation of NEAT1 lncRNAs in activated CD4 T cells to promote viral replication.	45,46
lncRNA ACOD1	Vesicular stomatitis virus (VSV), vaccinia virus (VACV), and herpes simplex virus type 1 (HSV-1)	NF-κB signaling	lncRNA ACOD1 increased the cytoplasmic enzymatic activity of the metabolic enzyme glutamic-oxaloacetic transaminase 2 (GOT2) and enhanced viral propagation in both human and animal cells. Viral replication was preserved by restoring GOT2 levels.	^47^
lncRNAs ISR, PAAN, and TSPOAP1	IAV	IFN-β activation; IFN-β serves as an IFN-stimulated gene	Enhanced IAV replication and altered IFN signaling	^48^^,^^49^
lncRNA NeST	Theiler's virus	Immune regulation	Triggered CD8 T cell IFN-γ locus activation	^50^
lncRNA-CD160	HBV	TNF-α and IFN-γ secretion inhibited CD8^+^ T cells	Prevented CD8 T cells from secreting IFN-γ and TNF-α by recruiting the histone-modification enzyme gene histone deacetylase 11 (HDAC11)	^51^
lncRNA Morrbid	Lymphocytic choriomeningitis mammarenavirus (LCMV)	PI3K/AKT pathway	Controlled CD8 T cell proliferation and effector activity by inhibiting the PI3K/AKT pathway	^52^
lncRNA NRON	Cytomegalovirus (CMV)	NFAT signaling pathway	Reduced expression of CD8 T cells in the elderly with persistent CMV infection	^53^
NKILA	HBV	NF-κB signaling pathway	Blocked binding between HBx and p65; NKILA reduced HBx-induced NF-κB activation; NKILA mutants that lacked critical regions for NF-κB suppression were unable to stop HBV replication.	^54^
lncRNA-CMPK2	HCV	Stimulation of IFN-α and IFN-γ	HCV increased replication by negatively regulating IFN-stimulated genes that code for proteins.	^55^
NRAV	IAV	Interferon-stimulated gene transcription suppression	Altered the interferon response to antivirals	^56^
NRON	HIV	NFAT-mediated expression	NRON modulated HIV's extensive host factor repertoire for infection and persistence.	^57^
lncRNA Dreh	HBV	HBx downregulation	Suppressed HBV-related HCC growth and metastasis by inhibiting vimentin expression both *in vitro* and *in vivo*	^58^
lncRNA#32	Encephalomyocarditis virus (EMCV)	IFN-stimulated gene expression regulation	lncRNA#32 controlled IFN-stimulated gene expression and EMCV viral propagation by binding to ATF2.	^59^
LncRNA zc3h7a	VSV	Significantly inhibited the antiviral innate immune response induced by RNA viruses recognized by RIG-I, such as VSV and SeV	Facilitated TRIM25-mediated K63-linked ubiquitination of RIG-I and downstream signaling; lncRNA zc3h7a deletion decreased the antiviral response and RIG-I signaling.	^60^
lncRNA MALAT1	HIV-1	promotes HIV-1 replication by epigenetically repressing the virus's silencing	Interaction with EZH2 eliminated epigenetic suppression of HIV-1 transcription by releasing EZH2 from the HIV-1 LTR promoter.	^61^

## Impact of lncRNAs on the host transcriptome and immune response

During viral infection, the host cell produces various lncRNAs to combat the infection. Similarly, viruses express a large number of lncRNAs to resist cellular antiviral activity. Recent studies suggest that lncRNAs regulate viral infections at the host-pathogen interface through innate immune responses, pathogen recognition receptor activation, epigenetic, transcriptional, and post-transcriptional modifications. Innate immunity is the initial line of defense against viral infections, in which viral RNAs are identified, inducing ISGs, and triggering proinflammatory responses during early infection stages. RNA or DNA virus infections regulate the expression of thousands of lncRNAs. Antiviral signaling via IFNs and TNF-α causes considerable differential expression of lncRNAs. These lncRNAs, in turn, modulate the host immune response by regulating pathogen recognition receptor (PRR)-related signaling, transcription factor translocation and activation, IFN and cytokine production, IFN-activated Janus kinase/signal transduction and transcription activation (JAK/STAT) signaling, and IFN-stimulated antiviral gene transcription.^62^

In addition to modulating the antiviral response, lncRNAs may act as unique diagnostic indicators and are potential targets in the development of new treatments. Virus-related lncRNAs released into serum may act as prognostic indicators.^63^ For example, two serum lncRNAs, uc001ncr and AX800134, show potential as new biomarkers for identifying HBV-positive HCC, particularly in the early stages of disease. The expression of lncRNA urothelial carcinoma-associated 1 (UCA1) and lncRNA WD repeat containing antisense to TP53 (WRAP53) was considerably higher in serum from HCC patients than in serum from those with chronic HCV infection or healthy volunteers. This finding indicates that lncRNA UCA1 and lncRNA WRAP53 overexpression may serve as new blood biomarkers for HCC diagnosis and prognosis.^64^ To summarize, lncRNAs are important regulators of transcriptional and post-transcriptional processes; hence, their functions in virus infection and therapy require further investigation.

Although thousands of lncRNAs are expressed during viral infection, there are only a few specific lncRNAs with experimentally proven functions in viral infection; therefore, the expression and functions of lncRNAs in viral infection need to be investigated further.^65^ A better knowledge of how the lncRNA transcriptome is altered in infected cells and how these changes affect the host-virus relationship should also be investigated. Such research may help identify novel cellular pathways involved in the antiviral response.

## lncRNAs involved in immunological dysregulation during viral infections

The genomes of prokaryotes and eukaryotes have been shaped by viral infections for millions of years. Because they are leftovers of germline infections that have developed into fixed forms, it is thought that endogenous retroviruses are responsible for half of the sequences in the human genome that are still present.^66^ To succumb to an infection and prevent it from dominating, the host needed to be both vulnerable to the virus and able to control its transmission. Moreover, a defense system providing these requirements must be self-limiting to return to homeostasis after viral clearance. Because it integrates both innate and adaptive responses, the immune system is one of the most effective defenses against dangerous extracellular pathogens. Research has demonstrated that numerous lncRNAs are actively expressed during immune responses, highlighting their potential roles in immune regulation. These lncRNAs exhibit a number of noteworthy mechanisms for triggering immune responses. In addition to examining lncRNAs in the host, another important field of study is host-pathogen interactions. Many ways of infection that affect their hosts have also been connected to lncRNAs. An AS lncRNA encoded by HIV-1 may help establish viral latency.^67^ Another lncRNA produced by the herpesvirus associated with Kaposi's sarcoma is polyadenylated nuclear RNA (PAN). PAN is restricted to its own genome, just as the AS lncRNA that HIV-1 encodes. However, PAN is responsible for initiating virtually all the transcription of the herpesvirus associated with Kaposi's sarcoma. By suppressing PAN, almost all transcription of the herpesvirus associated with Kaposi's sarcoma was shown to be stopped. Interestingly, PAN also impacts the host gene. The down-regulation of ribonuclease L (RNase L), IL-18, IFN, and interferon alpha 16 (IFNA16) was associated with heterochromatin formation, caused by PAN binding to PRC2 (particularly EZH2 and Suz12), much like some of the lncRNAs discussed earlier.^68^

The development and spread of HCC are significantly influenced by the immune system. This includes immune cell count changes, immune-related gene dysregulation, generation of chemokines and cytokines, and immune cell dysfunction.^69^ Viral infection requires both host lncRNA dysregulation and viral lncRNA expression. Proinflammatory activity and an antiviral reaction are induced by the host immune response to defend against viral infection.^70^^,^^71^ After infection with a virus, pathogen-associated molecular patterns (PAMPs) can be identified by Toll-like receptors (TLRs) and other PRRs, which trigger an innate immune response.^72^^,^^73^ RNA sequencing and microarray-based methods have identified lncRNAs related to TLR antiviral immune responses. For example, Carnero et al discovered that TLR4/TLR7-triggered lncRNA eosinophil granule ontogeny transcript (EGOT) enhanced viral multiplication and inhibited the antiviral response Click or tap here to enter text.^74^ Furthermore, considering the vital functions of TLR4 and TLR7 in host immunity and their interactions with transduction signaling pathways, including the NF-κB and IFN pathways, lncRNA EGOT may be involved in a range of activities during viral infection. Furthermore, it was revealed that a TLR2 ligand Pam 3 CSK 4 transcriptionally stimulated 62 lncRNAs and several protein-coding genes relevant to innate immunity.^75^ More research is needed to completely understand the precise downstream pathways of lncRNAs/TLRs in the antiviral immune response, in addition to identifying lncRNAs that are up-regulated in response to a TLR-induced viral response.^76^ Better understanding the fundamental processes by which lncRNAs influence signaling pathways and immune responses holds great promise for the development of targeted therapeutic approaches to combat these viral diseases.

## IFN-dependent and IFN-independent lncRNAs in viral invasion

IFNs, particularly type I interferon (IFN-I), are vital cytokines that control viral infection by activating ISGs.^77^ Although several host lncRNAs were identified that regulate ISG expression or are regulated by IFNs in viral infections, our understanding of the function of lncRNAs in infected cells remains limited, particularly regarding the mechanism by which viruses hijack the host metabolism for replication. Viruses regulate host metabolic networks to favor their survival. Most molecules that are responsive to viral infection and regulate these metabolic changes have not been identified, but are vital for understanding viral infection. Viruses also rely on host metabolic networks to complete their lifecycle.^78^

Recent studies demonstrated that, in response to viral infection or IFN, many lncRNAs are dysregulated and can affect viral replication in an IFN-dependent or -independent manner. Some viruses may hijack host lncRNAs to facilitate their replication and latency.^79^ Wang et al discovered that lncRNA ACOD1 promoted the reproduction of numerous viruses in both mouse and human cells. lncRNA ACOD1 is activated by various viruses but not by IFN-I and promotes viral replication in mouse and human cells. Impairing lncRNA ACOD1 (identified by its nearest coding gene Acod1) *in vivo* greatly reduced viral infection via IFN-I and IRF3-independent mechanisms. Cytoplasmic lncRNA ACOD1 was found to directly interact with the metabolic enzyme glutamic-oxaloacetic transaminase (GOT2) near the substrate niche, increasing its catalytic activity. Recombinant GOT2 protein and its metabolites rescued viral replication and boosted lethality in the absence of lncRNA ACOD1. This study identified a feedback loop of virus-induced lncRNA-mediated metabolic enhancement of viral infection as a possible target for developing broad-acting antiviral medicines.^33^

The involvement of lncRNAs in the host immune system during HBV infection has recently been investigated. lncRNA ENST00000519726 (lncRNA HEIM) was highly expressed in monocytes and increased during HBV infection. Elevated lncRNA HEIM expression was highly linked with the transforming growth factor (TGF) signaling pathway. Furthermore, changing the endogenous lncRNA HEIM quantity in monocytes had a substantial effect on TGF production.^80^ High lncRNA CD160 expression inhibited IFN-γ and TNF-α secretion in CD8^+^ T cells, reducing their immune response. lncRNA CD160 also interacted with histone deacetylase 11 (HDAC11) to create a complex on IFN-γ and TNF-α promoters, inhibiting their production. Thus, lncRNA CD160 was shown to act as an immunological suppressor. Indeed, knockdown of lncRNA CD160 prevented HBV infection.^51^

## lncRNAs related to HCC proliferation

Despite the complex and limited understanding of the hepatocarcinogenesis process, multiple studies have linked a large number of lncRNAs to the development of HCC.^81^ Host-derived lncRNAs, the expression of which is often disrupted due to viral infection, have been shown to function as scaffolds, decoys, signals, and guides to alter gene expression at the transcriptional, post-transcriptional, epigenetic, and even post-translational levels. The primary mechanisms of these lncRNAs include down-regulation of HBV replication and oncogene expression by the tumor suppressor. Only a small number of lncRNAs are recognized to diminish carcinogenesis, and the ones that do are normally down-regulated in HCC.^82^

By affecting downstream gene expression levels and inducing immune lymphocytes to initiate an inflammatory response. lncRNA regulates liver cancer cell activity in a variety of ways. Many lncRNAs are regulated by HBx, as demonstrated by numerous studies.^83^ HCC cells exhibit a range of biological activities as a result of these interactions between HBx-related lncRNAs and the cells, such as invasion, proliferation, apoptosis, and autophagy. The key pathways implicated include the phosphoinositide-3-kinase/protein kinase B (PI3K/Akt), TGF-β, mitogen-activated protein kinase (MAPK), p53, and Ca^2+^ pathways.^84^ lncRNAs have been shown to influence the expression of several critical genes involved in these pathways, including TLR9, STAT3, p53, TGF-β, and enhancer of zeste homolog 2 (EZH2). This affects HCC cell growth, which contributes to the development of HCC.^85^

The HBx protein can directly or indirectly bind to specific lncRNAs, including UCA1, deleted in lymphocytic leukemia 2 (DLEU2), HULC, and DBH antisense RNA 1 (DBH-AS1).^86^^,^^87^ Additionally, the levels of lncRNA in plasma from individuals with cured, dormant, or chronic HBV infections were shown to differ. In resolved cases, there was a significant increase in a few lncRNAs, such as HOXA transcript at the distal tip (HOTTIP), MEG3, and PCAT-32. These lncRNAs may serve as early predictors of HBV infection resolution.^88^ Furthermore, lncRNAs may affect the risk of HBV-related HCC in a number of ways. Therefore, investigating lncRNAs that are abnormally expressed in HBV-related HCC is an effective means of investigating the molecular process of this form of cancer.

Despite significant advances in surgical and diagnostic procedures, the outlook for HBV-related HCC remains poor. Blood indicators used to diagnose and predict HCC include des-γ-carboxy prothrombin (DCP), α-fetoprotein (AFP), and the lens culinaris agglutinin-reactive proportion of AFP (AFP-L3).^89^ However, the sensitivity and specificity of these tumor markers remain poor. This has motivated researchers to search for novel blood markers of HCC. Given that an increasing number of lncRNAs were found to affect the risk of HBV-related HCC, researchers have sought to determine if lncRNAs can serve as blood biomarkers for HBV-related HCC. Recently, the important functions of lncRNAs in the development and prognosis of HBV-related HCC have become more apparent through various methods, including loss- and gain-of-function studies and *in vitro* and *in vivo* evaluations. Numerous circulating lncRNAs and genetic variations of lncRNAs have been identified that may influence the risk and prognosis of HBV-associated HCC. Table 2 explains the lncRNAs implicated in the development of HCC. It is believed that these studies can shed light on the functions of lncRNAs to help prevent, diagnose, and treat HBV-related HCC.

**Table 2:** lncRNAs involved in HCC.

lncRNA in HCC	Role	Regulation	Reference
HULC	Autophagy	Up-regulated	^90^
MEG3	Inhibits tumors	Down-regulated	^91^^,^^92^
GAS5	Inhibits M2 macrophage polarization, drug resistance, and proliferation	Down-regulated	^93^^,^^94^
Dreh	Tumor suppressor	Down-regulated	
LINC00152	Boosts cell proliferation by controlling CCDN1	Up-regulated	^95^
UCA1	HCC apoptosis and proliferation	Up-regulated	^96^
MALAT1	Promotes aggressive tumor features and accelerates tumor growth	Up-regulated	^97^
LINC01093	Participates in LINC01093/miR-96-5p/ZFAND5/NF-κB signaling axis, which has an important role in the development of HCC	Down-regulated	^98^
LINC01194	Promotes HCC growth by reducing the expression of miR-655-3p	Up-regulated	^99^
HOTTIP	Linked to poor survival and metastasis formation	Up-regulated	^100^
MVIH	Inhibits cell apoptosis, promotes tumor development and intrahepatic metastasis, and engages in active angiogenesis	Up-regulated	^101^
H19	Induces resistance to drugs and encourages the invasion and growth of tumors; prevents HCC cell migration, invasion, and intrahepatic metastases	Up-regulated	^102^
lnc-DILC	Promotes liver cancer stem cell proliferation by blocking IL-6/JAK2/STAT3	Down-regulated	^103^
SNHG5	Promotes the growth and modulates characteristics of cancer stem cells in HCC by controlling the Wnt-signaling and UPF1 pathways	Up-regulated	^104^

## Immune escape and diagnostic potential of lncRNAs in HCC

By regulating downstream target gene expression and cancer-related signaling pathways, lncRNAs promote tumor cell proliferation, migration, invasion, autophagy, and apoptosis. Additionally, lncRNAs can be used as biomarkers to predict the efficacy of HCC treatment, including surgery, radiotherapy, chemotherapy, and immunotherapy, and potentially support individualized diagnostic and treatment tools. An increasing number of studies have demonstrated that serum lncRNAs, including ENSG00000258332.1, LINC00635, small nucleolar RNA host gene (SNHG1), lncRNA uc007biz.1 (LRB1), HULC, linc00152, and UCA1, serve as promising biomarkers for the early detection of HCC. Furthermore, the combined assessment of serum levels of these lncRNAs with AFP levels exhibited the highest sensitivity and accuracy for early diagnosis of HCC.^105^

Wang et al discovered that serum levels of LRB1 and levels of the protein AFP were clearly elevated in HCC patients, and that using LRB1 in conjunction with AFP as biomarkers significantly improved the diagnostic accuracy of HCC. Certain lncRNAs that are up-regulated in HCC can also be used as potential biomarkers for determining the stages of HCC tumors and overall survival. Additional studies revealed that serum LRB1 levels were positively correlated with HCC tumor stages and negatively associated with overall survival.^106^ Zheng et al discovered that serum UCA1 levels were elevated in HCC patients, and elevated serum UCA1 levels were associated with advanced tumor, node, and metastasis stage, higher tumor grade, and larger tumor size; therefore, UCA1 is a candidate marker for HCC diagnosis.^107^

## lncRNAs associated with HBV

Among the lncRNAs associated with HBV infection, most promote oncogenesis, whereas only a very small fraction prevents tumor formation. lncRNAs can influence HBV replication by blocking antiviral microRNAs, transporting transcription factors to the viral promoter, and removing epigenetic silencing from the virus's covalently closed circular DNA (cccDNA). According to reports, a number of HBV-induced lncRNAs interact with proteins such as p53, nucleophosmin 1 (NPM1), and phosphoglycerate kinase 1 (PGK1) to promote angiogenesis and cellular proliferation.^108^

lncRNA CD160 inhibited CD8 T cell secretion of IFN-γ and TNF-α during chronic HBV infection by targeting HDAC11, a histone-modifying enzyme. HDAC11 and lncRNA CD160 formed a copolymer that bound to TNF-α and IFN-γ promoters, causing aberrant chromatin assembly and H3K9Me1 methylation. Consequently, transcription and translation of TNF-α and IFN-γ are inhibited by lncRNA CD160. The lncRNA in dendritic cells (lnc-DC) is essential for dendritic cell proliferation, apoptosis, and immune responses through the TLR9/STAT3 signaling pathway. As part of the immune response triggered by HBV, lnc-DC decreased TNF-α, IL-6, IL-12, and IFN-γ levels, while increasing IL-1β levels in dendritic cells. lncRNA FTX dysregulation contributed to the abnormal activation of macrophages in HBV-induced cirrhosis, and reduced the expression of the inhibitory receptor Tim-3.^109^

## lncRNA interactions with the HBV HBx protein

The smallest open reading frame (ORF) of HBV, the HBx gene, encodes a regulatory protein composed of 154 amino acids. HBx was shown to participate in the development of HCC by trans-modulating numerous growth-regulating genes and activating multiple signaling pathways, such as NF-kB, p53, and Wnt signaling pathways. Recently published studies emphasized the important function of HBx in HBV-associated HCC. HBx, referred to as non-structural X, is one of the four overlapping ORFs of a protein that decodes the HBV genetic frames.^110^ Given that one study showed that HBx influenced the expression of genes linked to HCC, HBV infection has been directly associated with lncRNAs related to HCC.^111^

Multiple lncRNAs involved in viral immune responses, viral integration, and HBx regulation may influence HBV-associated HCC.^112^ Investigating lncRNAs that demonstrate differential expression in tissue samples from individuals with HBV-HCC may uncover new targets for diagnosis and treatment and elucidate carcinogenic pathways.^113^ Quantitative reverse transcriptase PCR and microarray studies indicated that HULC increased in HCC. HBx engaged with cAMP response element-binding protein (CREB) to enhance HULC expression in hepatoma cell lines and tissues from HBV-related HCC. HULC promoted the proliferation of hepatoma cells through the down-regulation of p18, a tumor suppressor gene located adjacent to HULC.^114^ HOTAIR and polo-like kinase 1 (Plk1) expression are stimulated by HBV replication, specifically HBx generation. Plk1 and HOTAIR worked together to contribute to epigenetic reprogramming linked to carcinogenesis.^115^ HBx-LINE1 suppressed miR-122, which was demonstrated to inhibit HBV replication by directly targeting the HBV pgRNA sequence. Therefore, by decreasing miR-122, HBx-LINE1 enhanced HBV replication.^116^^,^^117^

**Figure 2:**
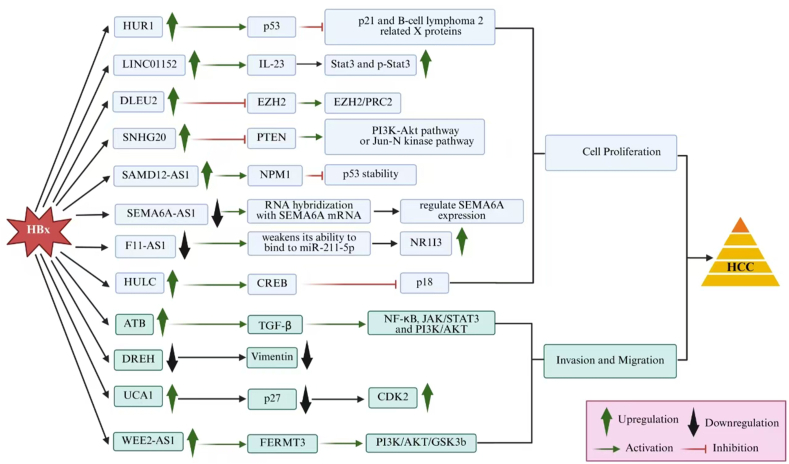
Regulatory networks of selected lncRNAs involved in the development of HBx-HCC.

The significance of lncRNAs in HBV replication is becoming increasingly clear. However, a large number of yet undiscovered lncRNAs, including those controlled by HBx, are likely required for HBV replication. In Figure 2, we briefly summarized lncRNAs regulated by HBx and their functions in the development of HCC. HBx is crucial for the transcription of the viral cccDNA minichromosome. Consequently, HBx controls HBV replication. The lncRNAs impacted by HBx may also influence HBV replication. The functions of these lncRNAs in HBV replication require additional research.

## Mechanism of lncRNAs' interactions with HBV and progression of HCC

Research has demonstrated that many lncRNAs associated with HBV and HCV infection influence gene expression, cellular growth, development, and apoptosis. To enhance our knowledge of HCC development, diagnosis, and treatment, it is essential to investigate the biological functions and regulation of lncRNAs associated with HCC.^118^ The involvement of lncRNAs in HBV pathogenesis and HCC development is thoroughly detailed in Table 3. The acetylation of p53 is essential for its role as a tumor suppressor. Lnc-Ip53 likewise competes with p53 for attachment to p300, preventing p300 from acetylating p53. These results indicated that the negative feedback loop of p53/lnc-Ip53 hampered p53 acetylation and function, facilitating HCC progression and chemoresistance.^119^, ^120^, ^121^

**Table 3:** lncRNAs associated with HBV and HCC proliferation.

lncRNA	Mechanism	Function	Reference
lncRNA FTX	lncRNA FTX/microRNA-545/Tim-3 plays a role in the inflammatory response of hepatitis B cirrhosis as it regulates Tim-3 gene transcription through the negative regulation of microRNA-545 expression and influences monocyte secretion of inflammatory cytokines.	HBV infection positively influenced lncRNA FTX, which could also be activated by HBx expression.	^122^^,^^123^
lncRNA SFMBT2	lncRNA SFMBT2 suppression reduced the level of HBV DNA in human hepatoma cells.	Enhanced expression of lncRNA SFMBT2 in inactive HBsAg carriers	^124^
lncRNA HUR1	Decreased levels of lncRNA HUR1 can restrict cell division, inhibit the transcriptional control of downstream genes such as p21 and B-cell lymphoma 2-associated X proteins; lnc-HUR1 binds to p53.	HBx enhanced lncRNA HUR1 transcription, which has a major impact on HBV-related HCC and may serve as a therapeutic indicator.	^125^
lINC01152	LINC01152 expression in HBV-positive patients. *In vitro*, HBx has been shown to significantly increase liver cancer cells and tissue. HBx increases the expression of LINC01152. Increases the levels of Stat3, p-Stat3, and IL-23.	Plays a crucial role in the development and progression of HBV-related HCC	^126^
lnc-DC	Knockdown of lnc-DC pSTAT3, TLR9, and SOCS3 levels, indicating the involvement of TLR9/STAT3 signaling.	Understanding the role of lnc-DC in DC development and immune response could reveal new mechanisms in HBV infection.	^127^
lncRNA DLEU2	HBx binds to the promoter of DLEU2; co-recruitment of HBx and DLEU2 on cccDNA replaced EZH2 on viral chromatin, increasing viral transcription and replication.	DLEU2 HBx binds to the target host promoter, inhibiting EZH2 and activating EZH2/PRC2 target genes in HBV-infected cells and HBV-related HCC.	^128^
lncRNA H19	lncRNA H19 reduced the expression of several crucial proteins in the epithelial–mesenchymal transition pathway, including N-cadherin, Vimentin, β-catenin, and MMP9. lncRNA H19 may regulate HBV-related HCC via the epithelial–mesenchymal transition pathway.	Significantly up-regulated in HBV-related HCC tissues	^129^
lncRNA SNHG20	The expression of SNHG20 in hepatoma cells shows a positive correlation with the HBx protein; HBx-SNHG20 controls cell growth and apoptosis.	The lncRNA SNHG20 is up-regulated in HBV-related HCC cells. HBx SNHG20 suppresses PTEN, leading to increased HCC cell proliferation and reduced apoptosis.	^130^
SAMD12-AS1	The overexpression of SAMD12-AS1 in HCC cells reduced p53 stability via the NPM1-HDM2-p53 pathway, thus affecting cell growth and apoptosis.	SAMD12-AS1 promotes cell proliferation and suppresses apoptosis.	^131^
SEMA6A-AS1	SEMA6A-AS1 might inhibit HBV-associated liver cancer through its interaction with SEMA6A mRNA and the regulation of its expression.	Lower levels of SEMA6AAS1 expression are associated with a negative prognosis in patients with HBV-related HCC.	^132^
F11-AS1	lncRNA F11-AS1 might influence downstream gene expression through its interaction with miR-211-5p.	The lncRNA F11-AS1/miR-211-5p/NR1I3 pathway is involved in the advancement of HBV-related HCC by disrupting the cellular physiological aspects of HCC.	^133^
WEE2-AS1	lncRNA F11-AS1 overexpression may boost NR1I3 expression by acting as a miR-211-5p′s ceRNA, eventually hindering the development of HBV(+). WEE2-AS1 and HBx up-regulated FERMT3 in HCC, which is linked to HBV infection in the liver.	The overexpression of WEE2-AS1 is associated with hepatic vascular invasion, low tumor differentiation, and unfavorable prognosis. The HBx-WEE2-AS1-FERMT3 pathway could be a potential therapeutic target for HCC related to HBV.	^134^
lncRNA ATB	Attaches to IL-11 mRNA and stimulates STAT3 signaling, enhancing histochemistry in metastatic tumor cells; fosters the invasion-transfer cascade reaction. These results indicate that lncRNA-ATB, a mediator of TGF-β signaling, might serve as a therapeutic target for HCC metastasis.	HBx triggers autophagy and enhances the expression of TGF-β and lncRNA-ATB.	^135^^,^^136^
lncRNA N335586	lncRNA n335586 associated with miR-924 to enhance the expression of the host gene CKMT1A, resulting in increased migration and invasion of liver cancer cells; may contribute to the advancement of HBV-related liver cancer by affecting cell movement and invasion.	Deep sequencing identified lncRNA N335586 as one of the highest lncRNAs in liver tumors associated with HBV.	^137^
N346077	N346077 prevented the invasion and movement of liver cancer cells.	One of the most down-regulated lncRNA in HCC cells	^138^
DREH	DREH is bound to Vimentin and suppresses its expression, altering the cytoskeletal structure and hindering tumor metastasis.	It inhibits cell migration and suppresses tumor growth in HBx-mediated liver cancer.	^139^
AX800134	Recognized as a potential new biomarker for diagnosing HCC; AX800134 was discovered to enhance HBV-related HCC and exhibit a specific mechanism of action.	AX800134 emerged as a novel possible biomarker for diagnosing HCC.	^140^
lncRNA MEG3	The levels of lncRNA MEG3 are associated with liver fibrosis in patients with CHB. The expression of serum lncRNA MEG3 was reduced in patients with chronic hepatitis B and was negatively associated with liver fibrosis.	Serum biomarker for diagnosing CHB	^141^

lncRNA HOXD-AS1, which is elevated in HCC and influenced by STAT3, was shown to enhance the expression of SRY-related high-mobility-group box 4 (SOX4) by competitively interacting with miR-130a-3p and activating two direct SOX4 targets, EZH2 and matrix metallopeptidase 2 (MMP2). Thus, HCC metastasis was enhanced.^142^ The lncRNA CSMD1-1, also elevated in HCC, was shown to enhance MYC signaling and promote HCC development by specifically binding to MYC to inhibit its ubiquitination and subsequent degradation.^143^

TGF-β exhibits both pro- and anti-cancer activity in the pathophysiology of HCC. The propagation of HCC is promoted by a positive feedback mechanism that includes TGF-β, small mothers against decapentaplegic (SMAD), and lncRNA urinary transforming growth factor (lnc-UTGF). TGF-β was found to enhance lnc-UTGF transcription by binding to the lnc-UTGF promoter through SMAD2/3 and subsequently engaged and stabilized SMAD2/4 mRNA. This feedback loop enhanced HCC metastasis by boosting TGF-β signaling.^144^ In HCC, elevated lncRNA pituitary tumor-transforming 3 pseudogene (PTTG3P) levels were linked to the expression of pituitary tumor-transforming gene 1 (PTTG1). In HCC cells, increased PTTG1 levels and activation of the PI3K/AKT pathway stimulated the G1/S phase transition, enhanced cell proliferation, and promoted epithelial–mesenchymal transition (EMT). PTTG3P likewise stimulates the PI3K/AKT pathway.^145^ Yin Yang 1 (YY1) was reported to bind to the cancer susceptibility 11 (CASC11) promoter of the lncRNA, enhancing lncRNA expression in HCC. To accelerate HCC development, CASC11 additionally recruited eukaryotic translation initiation factor 4A3 (EIF4A3), which boosted early 2-factor 1 (E2F1) expression and activated the PI3K/AKT/mTOR and NF-κB pathways. The suppression of CASC11 resulted in decreased levels of programmed cell death-Ligand 1 (PD-L1), indicating a connection between CASC11-driven HCC progression and PD-L1-facilitated immune evasion.^146^ The exploration of the transcribed-ultra conserved region (T-UCR) downstream of the Wnt/β-catenin pathway in HCC resulted in the identification of the lncRNA T-UCR uc.158. Activation of T-UCR through Wnt signaling might play a role in the development and progression of HCC. Uc.158 was demonstrated to be uniquely activated in cancer cells, suggesting a specific role for it in Wnt signaling and a potential therapeutic target.^147^

## Emerging tools in lncRNA detection

lncRNAs are a significant and functionally varied component of the transcriptome, regulating gene expression and cellular functions in a variety of biological environments. Clustered regularly interspaced short palindromic repeats (CRISPR)-based screening methods have revolutionized the study of lncRNAs by enabling precise and scalable changes in their expression. CRISPR technology, RNA interference techniques, and overexpression systems can confirm lncRNA roles in cellular models with remarkable clarity.^148^ These orthogonal approaches are critical for expanding our understanding of lncRNA biology and hastening their development as biomarkers and therapeutic targets. By combining high-resolution sequencing with functional perturbation approaches, researchers may deconstruct the regulatory networks controlled by lncRNAs.^149^

With its broad dynamic range and high sensitivity, RNA sequencing remains the primary method for identifying and quantifying lncRNAs. RNA sequencing enables researchers to profile the entire transcriptome without requiring pre-designed probes or prior knowledge of gene annotations. RNA sequencing can also detect alternatively spliced transcripts of lncRNAs to reveal the complexity and diversity of lncRNA species that may have different functions.^150^

A better understanding of lncRNAs will broaden the genomic landscape and shed light on certain complex biological mechanisms, thus identifying new therapeutic targets. However, many of these efforts have been dwarfed by the complexity of the lncRNA world, most likely because of the low expression and naturally high expression variability.^151^ Given the abundance of available data resources, genome-scale research will be invaluable for advancing lncRNA studies. Identifying novel lncRNAs is the foundation for lncRNA studies. A complete and comprehensive genomic annotation, which refers to the full genomic architecture and boundary information, is the cornerstone of the follow-up to functional and mechanistic studies. Current approaches to identifying novel transcripts at the genome scale are based on numerous raw RNA-sequencing data combined with bioinformatic analysis pipelines containing alignments, assembly, quantification, and difference analyses, and constantly emerging systematic bioinformatic technologies continue to provide more accurate and comprehensive annotations.^152^

## Challenges in lncRNA detection

The advancement of high-throughput RNA sequencing technology has resulted in the identification of hundreds of noncoding RNA genes. The secrets of lncRNAs are gradually revealed. With the advancement of lncRNA research, particularly with a better understanding of the association between lncRNA and HCC, an increasing number of lncRNAs have been investigated and found to be useful for the early diagnosis of HCC.^153^

Because of their noncoding nature and different functions, lncRNAs require a systematic approach to determining their specific roles in HCC drug resistance. CRISPR/CRISPR-associated protein 9 (Cas9) screening is an emerging strategy to search for possible important genes or therapeutic targets through deletions or activations.^154^ Several technical difficulties impede the proper detection and evaluation of lncRNAs in HBV-related HCC. These include difficulties in reliably detecting and measuring lncRNAs due to their low expression levels and structural complexity, constraints in analyzing their various activities and subcellular localization, and difficulties in developing reliable biomarkers for early detection and prognosis.^155^

## Conclusion

Numerous lncRNAs are either up-regulated or down-regulated following viral infection, indicating their potential involvement in the host's antiviral response. Their varying expression levels further suggest that lncRNAs may function as prognostic indicators or have implications for therapeutic diagnostics. An effective early diagnostic method for viral infections may be the discovery of lncRNA biomarkers in patient serum. In addition to the dysregulation of host lncRNAs, viral infection also requires the expression of viral lncRNAs. Even though thousands of lncRNAs are expressed during viral infection, more research is still needed to understand the functions and actions of these lncRNAs in viral infection. This is due to the fact that very few distinct lncRNAs have been shown to have roles in experiments. Examining the manner in which an infected cell's lncRNA transcriptome is altered and how these changes affect the host-virus interaction may also help decipher the lncRNA transcriptome. Such research could help identify new cellular pathways involved in the antiviral response. The currently available data clearly demonstrate the essential functions that lncRNAs have in transcription, replication, and immunity during the antiviral response. Clarifying the function of virus-regulated lncRNAs that have been experimentally confirmed still requires additional research. In summary, an improved understanding of the functions of lncRNAs in viral illnesses may ultimately lead to the development of new treatment approaches. Although diagnostic and treatment strategies for HCC are rapidly evolving, such as new interventional chemotherapies, molecular targeted therapy, and liver transplantation, overall survival rates for liver cell cancer patients remain disappointing. There is an urgent need to find new therapeutic targets and improve the overall survival of HCC patients. As lncRNAs are a new class of regulatory molecules that modulate gene expression at the transcriptional, posttranscriptional, and epigenetic levels, and affect the proliferation, apoptosis, invasion, and metastasis of HCC cells, they provide a new direction for the development and treatment of HCC.

## CRediT authorship contribution statement

**Faisal Mahmood:** Writing – review & editing, Writing – original draft, Validation, Investigation, Funding acquisition. **Maher Un Nisa Awan:** Writing – review & editing, Writing – original draft. **Mengting Luo:** Writing – review & editing, Writing – original draft, Investigation. **Xiao Li:** Writing – review & editing, Writing – original draft, Investigation. **Xiaobin Peng:** Writing – review & editing, Writing – original draft. **Jun Xu:** Writing – review & editing. **Jia Wei:** Writing – review & editing, Validation, Supervision, Resources. **Taicheng Zhou:** Validation, Supervision, Resources, Funding acquisition.

## Funding

This work was supported by the National Foreign Expert Project (China) (No. Y20240212), Leading Talents in Medical Disciplines of Yunnan Province, China (No. D-2024002), Reserve Talents for Middle, Young aged Academic and Technical Leaders in Yunnan Province, China (No. 202205AC160023), The 14th Five-Year Plan Yunnan Provincial Key Clinical Specialty Project - Infectious Diseases Department (China) (No. ZKF2025015), Yunnan Provincial Major Science and Technology Special Project Plan – Biomedicine Special Project (China) (No. 202502AA310011), Yunnan Fundamental Research Projects (China) (No. 202301AT070248), and Kunming Medical Joint Project of Yunnan Provincial Department of Science and Technology (China) (No. 202401AY070001-173, 202501AY070001-216, 202201AY070001-275).

## Conflict of interests

The authors declared no conflict of interests.
